# Evaluation of ferritin and TfR level in plasma neural-derived exosomes as potential markers of Parkinson’s disease

**DOI:** 10.3389/fnagi.2023.1216905

**Published:** 2023-09-19

**Authors:** Zhi-ting Chen, Chu-zhui Pan, Xing-lin Ruan, Li-ping Lei, Sheng-mei Lin, Yin-zhou Wang, Zhen-Hua Zhao

**Affiliations:** ^1^Department of Neurology, Fujian Medical University Union Hospital, Fuzhou, Fujian, China; ^2^Shengli Clinical Medical College of Fujian Medical University, Fuzhou, Fujian, China; ^3^Department of Neurology, Fujian Provincial Hospital, Fuzhou, Fujian, China

**Keywords:** biomarker, ferritin, transferrin receptor, exosome, Parkinson’s disease

## Abstract

**Introduction:**

Early diagnosis of Parkinson’s disease (PD) remains challenging. It has been suggested that abnormal brain iron metabolism leads to excessive iron accumulation in PD, although the mechanism of iron deposition is not yet fully understood. Ferritin and transferrin receptor (TfR) are involved in iron metabolism, and the exosome pathway is one mechanism by which ferritin is transported and regulated. While the blood of healthy animals contains a plentiful supply of TfR positive exosomes, rare study has examined ferritin and TfR in plasma neural-derived exosomes in PD.

**Methods:**

Plasma exosomes were obtained from 43 patients with PD and 34 healthy controls. Neural-derived exosomes were isolated with anti-human L1CAM antibody immunoabsorption. Transmission electron microscopy and western blotting were used to identify the exosomes. ELISAs were used to quantify ferritin and TfR levels in plasma neural-derived exosomes of patients with PD and controls. Receivers operating characteristic (ROC) curves were applied to map the diagnostic accuracy of ferritin and TfR. Independent predictors of the disease were identified using logistic regression models.

**Results:**

Neural-derived exosomes exhibited the typical exosomal morphology and expressed the specific exosome marker CD63. Ferritin and TfR levels in plasma neural-derived exosomes were significantly higher in patients with PD than controls (406.46 ± 241.86 vs. 245.62 ± 165.47 ng/μg, *P* = 0.001 and 1728.94 ± 766.71 vs. 1153.92 ± 539.30 ng/μg, *P* < 0.001, respectively). There were significant positive correlations between ferritin and TfR levels in plasma neural-derived exosomes in control group, PD group and all the individuals (rs = 0.744, 0.700, and 0.752, respectively). The level of TfR was independently associated with the disease (adjusted odds ratio 1.002; 95% CI 1.000–1.003). ROC performances of ferritin, TfR, and their combination were moderate (0.730, 0.812, and 0.808, respectively). However, no relationship was found between the biomarkers and disease progression.

**Conclusion:**

It is hypothesized that ferritin and TfR in plasma neural-derived exosomes may be potential biomarkers for PD, and that they may participate in the mechanism of excessive iron deposition in PD.

## Introduction

Parkinson’s disease (PD) is the second most common neurodegenerative disease, characterized by a set of extrapyramidal motor features. Progressive degeneration of dopamine (DA) neurons in the substantia nigra pars compacta and aggregation of α-synuclein and formation of Lewy bodies are its typical pathological manifestations (Berardelli et al., 2013).

The diagnosis of PD mainly relies on typical clinical symptoms such as bradykinesia, muscular rigidity, resting tremor, and/or postural instability. However, in the early stages of the disease, the motor symptoms may not yet occur, or they may be atypical, making diagnosis challenging. Hence, early diagnosis of PD remains difficult. To support the diagnosis, there have been increasing research efforts to evaluate the diagnostic performance of magnetic resonance imaging (MRI) using iron sensitive sequences targeting the substantia nigra (SN) to distinguish patients with PD from control participants (Calloni et al., 2018). These studies are based on theory that abnormal brain iron metabolism results in excessive iron deposition in PD.

Iron metabolism disorder and iron-mediated oxidative stress are believed to be important pathological mechanisms underlying the degeneration of DA neurons. Increased iron levels have been observed in the substantia nigra in post-mortem studies of PD and *in vivo* using transcranial sonography, susceptibility weighted imaging (SWI), and quantitative susceptibility mapping (Groger and Berg, 2012; Langkammer et al., 2012). However, the mechanism of iron deposition is still unclear.

Ferritin and transferrin receptor (TfR) are involved in iron metabolism. Ferritin not only regulates cell iron content and protect organisms from iron toxicity, but also transports iron through regulation (Truman-Rosentsvit et al., 2018). Studies have shown that exosomes are one of the non-classical mechanisms of ferritin secretion and transport regulation. Recent studies have also found that after binding to TfR, ferritin can cross the blood-brain barrier through transendocytosis of brain endothelial cells, rather than being trapped in lysosomes (Fan et al., 2018). The blood of healthy animals contains plenty of TfR-positive exosomes.

Studies of iron and iron-related proteins levels in cerebrospinal fluid (CSF), serum, plasma, and urine have shown conflicting results. Meta-analyses of these studies have revealed that CSF and serum/plasma ferritin and transferrin concentrations did not differ significantly between PD patients and controls (Jimenez-Jimenez et al., 2021). However, recent studies have shown that exosomes were a pathway for neurons to divert proteins from neurons into the CSF or into the peripheral blood via the blood-brain barrier (Shi et al., 2014). Exosomal α-synuclein in neural-derived blood exosomes was found to be increased in patients with PD (Shi et al., 2016). Chalwa’s group studied the iron homeostasis in restless legs syndrome, and found that the patients had higher levels of total ferritin and heavy-chain ferritin, and similar levels of TfR in serum neural-derived exosomes (Chawla et al., 2019). To investigate the mechanism of iron deposition and to identify potential novel biomarkers for PD, this study evaluated the levels of ferritin and TfR level in plasma neural-derived exosomes using enzyme-linked immunosorbent assay (ELISA).

## Materials and methods

### Participants

This study was conducted at the Department of Neurology, Fujian Provincial Hospital, Fuzhou, China. The laboratory technicians and statistical analysts were blinded to the clinical information and grouping. The study was approved by the Ethics Committees of Fujian Provincial Hospital, and written informed consent was obtained from all participants.

From January 2020 to June 2021, 43 PD patients were recruited based on the movement disorder society clinical diagnostic criteria for Parkinson’s disease (Postuma et al., 2015). The control group consisted of 34 healthy community volunteers who showed no signs or symptoms of neurological disease. All participants were excluded based on the following criteria: (1) presence of tumors, (2) secondary parkinsonism, Parkinson-plus syndrome, or other neurodegenerative diseases, (3) severe craniocerebral trauma, (4) any inflammatory, infectious, or autoimmune diseases, and (5) severe systemic diseases, such as anemia, hepatosis, heart failure, pulmonary disorders, and chronic renal failure.

Medical history, physical and neurological examinations, and laboratory tests were conducted on all participants. Blood routine and aminopherase tests were normal for all participants. The severity of PD was assessed using Hoehn–Yahr (H–Y) scores and the disease course was recorded for the PD patients. Fasting blood samples were collected from all individuals in the morning, and plasma was separated by centrifugation at 2,000 × *g* for 20 min. The aliquots were stored frozen at −80°C.

### Isolation of plasma neural-derived exosomes

**FIGURE 1:**
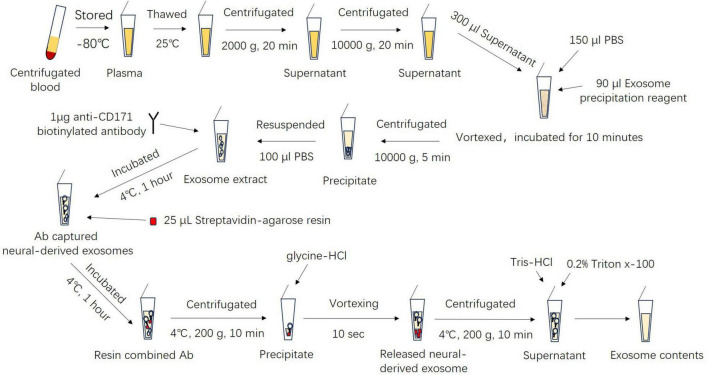
Flowchart outlining the experimental procedures described in this figure. Ab, antibody.

Exosomes were isolated following the experimental protocol outlined in our previous report (Zhao et al., 2018). The flow chart of the experimental procedure is shown in Figure 1. Initially, plasma exosomes were isolated using the Total Plasma Exosome Isolation Kit (Invitrogen, USA) as per the manufacturer’s instructions. The frozen sample was thawed in a 25°C water bath, followed by centrifugation at 2,000 × *g* at room temperature for 20 min to remove cells and debris. Next, the supernatant was centrifuged again at 10,000 × *g* at room temperature for 20 min to further remove debris. A total of 300 μL of the resulting supernatant was transferred to a new tube and mixed with 150 μL of PBS. The exosome precipitation reagent was added in a 0.2:1 ratio and the solution was vortexed until homogenous. The mixture was incubated for 10 min at room temperature, then centrifuged at 10,000 × *g* at room temperature for 5 min. The supernatant was removed, and the precipitate was resuspended in 100 μL of PBS containing inhibitor cocktails and 100 μL of 3% bovine serum albumin. Subsequently, the isolation neural-derived exosomes from plasma were performed. The suspensions were incubated at 4°C for 1 h with 1 μg of mouse anti-human CD171 biotinylated antibody (clone 5G3, eBioscience, USA) for combining the neural cell adhesion molecule L1 expressing specifically in neuron membranes, and 25 μL of streptavidin-agarose resin (Thermo Scientific, USA) with 50 μL of 3% BSA for combining biotin in anti-CD171 biotinylated antibody, respectively. After centrifugation at 4°C for 10 min at 200 × *g* and removal of the supernatant, each precipitation containing the neural-derived exosomes was collected, then suspended in 50 μL of 0.05 M glycine-HCl (pH 3.0) by vortexing for 10 s to separate the exosomes from the resin. After that, the former was relieved into the supernatant. Subsequently, 50 μL of 3% BSA was added, and the mixture was centrifuged at 200 × *g* for 10 min at 4°C. The resulting supernatants were transferred to clean tubes containing 5 μL of 1 M Tris-HCl (pH 8.0) and mixed to neutralize the acidity of glycine-HCl. Finally, 150 μl of PBS with 0.2% Triton x-100 and inhibitor cocktails was added to each suspension and incubated for 10 min at room temperature to rupture the membrane of the exosomes. The samples were then stored at −80°C until use. The protein concentration of the sample was determined using the Bradford protein assay (Tiangen Biotech, China), with bovine serum albumin as a standard.

### Transmission electron microscopy

The exosome extract was applied to a sealing membrane, and a copper grid (200 mesh, Zhongjingkeyi Technology Co., Ltd., China) with the front side facing the liquid was placed on the droplet. The grid was left for 2 min to absorb the exosome extract, before being transferred to a solution of 40 g/L uranyl acetate that was dripped onto the sealing membrane for negative staining. The front side of the grid was facing the droplet and left in the dark for another 2 min. Excess liquid was absorbed with filter paper, and the results were observed using a Tecnai SPIRIT transmission electron microscope (FEI, USA) operating at 120 kV.

### Western blotting

A volume of 20 μL of lysed samples were separated on 10% sodium dodecyl sulfate-polyacrylamide gels and transferred onto a nitrocellulose membrane. The membrane was blocked using 3% skim milk before incubation with primary antibodies, specifically mouse anti-CD63 (1:500; Servicebio, China). Next, the membrane was incubated with goat anti-mouse IgG antibodies conjugated to horseradish peroxidase (1:5,000; Servicebio, China) and visualized using a chemiluminescence reagent (Servicebio, China). The immunoreactivity was detected on a Bio-Rad ChemiDoc MP (Bio-Rad, USA).

### ELISA quantification of exosome proteins

The protein content of plasma neural-derived exosomes was measured using ELISA kits for human ferritin (Catalog Number: DY3541-05) and human TfR (Catalog Number: DY2474) (R&D System, USA), following the manufacturer’s protocol. Each test was repeated twice for both human ferritin and human TfR. The total protein of the sample served as the control.

### Statistical analysis

All statistical analyses were performed using SPSS Statistics 23.0 (IBM, USA). A *p*-value < 0.05 was considered statistically significant in all cases. Continuous variables, including age, duration of disease, and levels of human ferritin and human TfR in exosomes, were presented as mean ± standard deviation (SD). Differences between sexes were compared using a χ^2^ test. A *t*-test was used to identify statistically significant differences when the data were normally distributed, and a Mann–Whitney U-test was used when the data did not conform to the normal distribution. Pearson’s correlation coefficients were calculated to evaluate correlations among biomarkers for human ferritin and human TfR. In cases where the data did not conform to a normal distribution, Spearman’s correlation coefficients were obtained to evaluate the correlations among the biomarkers. Receiver operating characteristic (ROC) curves were generated to evaluate the sensitivities and specificities for the biomarkers in differentiating PD from healthy controls. The optimal cut-off value for an ROC curve was defined as the point associated with the maximal sum of sensitivity and specificity. Binary logistic regression model assessed associations between biomarkers and the disease.

## Results

A total of 43 PD patients and 34 healthy individuals were included in this study, comprising 26 males and 17 females in the PD group, with an average age of 66.6 ± 9.6 years old. The PD group had a mean disease duration of 4.4 ± 3.5 years, and an H–Y stage of 2.7 ± 1.2. The control group comprised 17 males and 17 females with the average age of 63.7 ± 10.0 years old (Table 1).

**TABLE 1:** Baseline data and comparation of the level of ferritin and TfR in two groups.

	Control group	PD group			*P* 1	*P* 2
		Early-stage	Advanced-stage	total		
*n*	34	16	27	43	–	–
Age						
mean ± SD	63.7 ± 10.0	64.5 ± 10.8	67.9 ± 8.8	66.6 ± 9.6	0.202^a^	0.203^b^
range	40–80	43–84	42–82	42∼84	–	–
Sex (male/female)	17/17	7/9	19/8	26/17	0.358	0.084
Duration of disease (years)		2.3 ± 2.5	5.6 ± 3.8	4.4 ± 3.5	–	< 0.001^b^
H–Y stage		1.4 ± 0.5	3.5 ± 0.8	2.7 ± 1.2	–	< 0.001^b^
Ferritin (ng/μg)	245.62 ± 165.47	385.00 ± 203.52	419.17 ± 264.85	406.46 ± 241.86	0.001^a^	0.802^b^
TfR (ng/μg)	1,153.92 ± 539.30	1,758.85 ± 527.24	1,711.22 ± 887.89	1,728.94 ± 766.71	< 0.001^b^	0.248^b^

^a^Student’s *t*-test.

^b^Mann–Whitney U-test. *P* 1, compare between control subjects and PD; *P* 2, compare between early-stage PD and advanced-stage PD.

**FIGURE 2:**
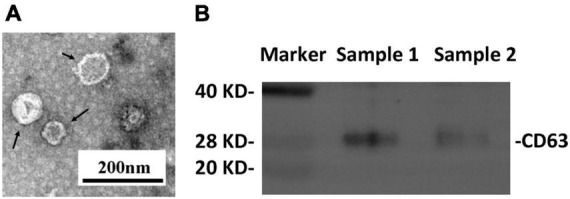
Identification of plasma neural-derived exosomes. Representative transmission electron microscopy image showing the morphological characteristics of the exosomes (black arrows) **(A)**. Western blot images **(B)** showing that the exosomal marker CD63 was expressed in exosomal samples.

**FIGURE 3:**
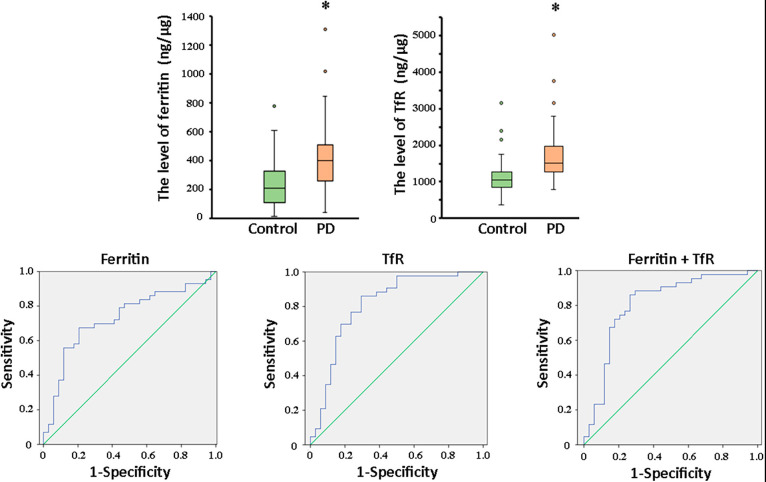
Comparation of ferritin and TfR in PD and controls, and ROC analysis of biomarkers for PD diagnosis. The box plots showed the comparison of the levels of ferritin **(A)** and TfR **(B)** in the two groups. *, *P* < 0.01. In the whole cohort, ferritin in plasma neural-derived exosomes provided an AUC of 0.73 (sensitivity = 67.4%, specificity = 79.4%) for PD versus controls **(C)**. TfR performed similarly (AUC = 0.812, sensitivity = 86.0%, specificity = 70.6%) in the whole cohort **(D)**. And, the combination of ferritin and TfR did not obviously improve the performance with AUC of 0.808 (sensitivity = 86.0%, specificity = 73.5%) **(E)**.

Transmission electron microscopy and western blot analysis were used to confirm the presence of plasma neuronal-derived exosomes, which exhibited the typical exosomal morphology (Figure 2A) and expressed CD63, a specific exosome marker (Figure 2B). The levels of ferritin and TfR in plasma neural-derived exosomes were then quantified using ELISA, with each sample analyzed in duplicate. The levels of ferritin in the PD group were significantly higher than those in the control group (406.46 ± 241.86 vs. 245.62 ± 165.47 ng/μg, *P* = 0.001) (Figure 3A), and the levels of TfR in the PD group were also significantly higher than those in the control group (1,728.94 ± 766.71 vs. 1,153.92 ± 539.30 ng/μg, *P* < 0.001) (Figure 3B). The most parsimonious model retained TfR as an independent predictor for PD (Table 2), with an increased risk of adjusted odds ratios of 1.002 (95% CI 1.000∼1.003).

**TABLE 2:** Binary logistic regression analysis results of PD.

	*B*	*Exp (B)*	95% CI	*P*-value
Ferritin	0.001	1.001	0.998∼1.005	0.452
TfR	0.002	1.002	1.000∼1.003	0.039*

*, *P* < 0.05.

Receivers operating characteristic (ROC) analysis was conducted to assess the diagnostic potential of ferritin and TfR in plasma neural-derived exosomes for PD. The sensitivity and specificity of both biomarkers were determined, and their ROC performance was moderate. The area under curve (AUC) for ferritin was 0.730 (Figure 3C) with a sensitivity of 67.4% and specificity of 79.4% at a cutoff value of 331.80 ng/mg. The AUC for TfR was 0.812 (Figure 3D) with a sensitivity of 86.0% and specificity of 70.6% at a cutoff value of 1,184.18 ng/mg. The AUC for the combination of ferritin and TfR was 0.808 (Figure 3E). It’s sensitivity and specificity were 86.0 and 73.5%, respectively, with a cutoff value of 0.35 on the predicted risk algorithm. The combination of ferritin and TfR did not enhance the performance of discrimination significantly.

**FIGURE 4:**
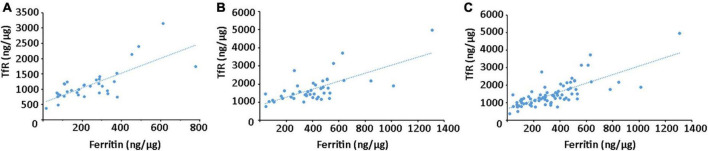
Correlation analysis between ferritin and TfR in plasma neural-derived exosomes. A significant positive correlation between ferritin and TfR was observed in controls (rs = 0.744, *p* < 0.001) **(A)**, patients with PD (rs = 0.700, *p* < 0.001) **(B)**, and all the individuals (rs = 0.752, *p* < 0.001) **(C)**.

Furthermore, a correlation analysis was conducted to evaluate the relationships between ferritin and TfR levels in plasma neural-derived exosomes. A significant positive correlation was observed between ferritin and TfR levels in both healthy controls (Figure 4A) and PD patients (Figure 4B). This finding was consistent across all subjects in this study (Figure 4C).

To investigate the potential relationship between plasma neural-derived exosome levels of ferritin and TfR and disease severity/progression, we conducted a correlation analysis. In line with the disease stage, the PD patients in our study were categorized into two groups: 16 patients at the early stage of PD (H–Y 1 to 2) and 27 patients at the advanced stage (H–Y 3 to 5). Our results showed no significant difference in the levels of ferritin and TfR between the early-stage group and the advanced-stage group (385.00 ± 203.52 vs. 419.17 ± 264.85 ng/μg, *P* = 0.802 and 1,758.85 ± 527.24 vs. 1,711.22 ± 887.89, *P* = 0.248, respectively) (Table 1). Moreover, we found no significant correlation between the levels of ferritin or TfR and disease duration or H–Y stages (*P* = 0.274 and 0.802 for ferritin, and *P* = 0.875 and 0.453 for TfR, respectively).

## Discussion

In this study, we extracted neural-derived exosomes from plasma and detected the levels of ferritin and TfR. Both of them were significantly higher in patients with PD than normal individuals. Further analysis showed that TfR was as an independent predictor for PD. The diagnostic potential of ferritin and TfR were moderate for PD.

The diagnosis of PD is based upon established clinical criteria that primarily rely on the presenting symptoms. Although there has been some progress in the study of biofluid biomarkers and neuroimaging in PD (Eusebi et al., 2017), available and effective biomarkers remain an urgent need for the disease. Blood is always the most intriguing sample source. It is easier to obtain than CSF, without repeated lumbar punctures. There is still no recognized standard for neuroimaging, and corresponding procedures need to be set according to different MRI instruments. Prolonged immobilization during the examination also limited some patients.

In the field of diagnostic biomarkers, molecules related to the pathophysiological mechanisms occurring in the disease, such as α-synuclein species (Parnetti et al., 2019) and iron metabolism-related proteins, have been believed as the most promising targets. α-synuclein oligomers and iron deposits in the substantia nigra pars compacta play key roles in the pathogenesis of PD (Martin et al., 2008). When the iron concentration surpasses the transport capacity of transferrin, the excess iron initiates a redox process in metabolism that produces hydroxyl free radicals (Youdim and Riederer, 1993). These free radicals may cause pathological alterations such as cell damage, lipid peroxidation, mitochondrial dysfunction, chronic inflammation, and α-synuclein deposition (Gutteridge, 1994; Sian-Hulsmann et al., 2011), which are all believed to contribute to neurodegenerative diseases. Iron deposition can lead to conformational changes in α-synuclein, which can subsequently lead to the production of α-synuclein oligomers (Dehay et al., 2016; Henrich et al., 2018). Autopsies and iron-sensitive sequences in MRI of PD patients have shown an increase in iron deposition in the substantia nigra (Griffiths et al., 1999; Sugiyama et al., 2018). Interestingly, studies of other neurodegenerative diseases have also suggested that iron metabolism disorder is associated with disease progression. Similarly, studies of other synuclein diseases, such as multiple system atrophy, have also found that iron deposition is involved (Dickson et al., 1999; Galvin et al., 2000). In some cohort studies about Alzheimer’s disease, the researchers observed that the level of CSF ferritin might accelerate the disease process (Ayton et al., 2017, 2018; Pan et al., 2022), and relate to phosphorylated tau, total tau, and inflammatory factors (Pan et al., 2022).

Ferritin exists in the cytoplasm of almost all cells. It is a 24-polymer protein composed of two subunits and mainly plays the biological function of storing iron. The relationship between serum ferritin and PD remains inconclusive. In a meta-analysis of 8 studies comprising 635 PD patients and 444 controls found a significant increase in serum or plasma ferritin levels (Wei et al., 2018). In contrast, another meta-analysis of 27 studies, including 2,006 patients and 2,098 controls, found that serum or plasma ferritin levels were comparable between PD patients and control groups (Jimenez-Jimenez et al., 2021). Although the inclusion criteria and methodology of PD patients varied across studies, resulting in substantial heterogeneity in the combined results, both studies reported similar serum transferrin levels in PD patients and controls.

Notably, since mammalian ferritin lacks signaling peptides that regulate classical Golgi secretion, extracellular secretion of ferritin is mainly achieved through non-classical protein secretion pathways, secretory autophagy (Cohen et al., 2010) and exosome pathway (Truman-Rosentsvit et al., 2018). Ferritin in exosomes is shielded by exosomal lipid bilayer membrane and is not easily affected by the peripheral environment, which may better reflect the situation of iron metabolism *in vivo*. Therefore, ferritin in exosomes may more accurately reflect intracellular iron storage, and may also be involved in the distribution of iron between cells, so as to play an important role in the maintenance of brain iron homeostasis. So far, there has been no study on ferritin in plasma neural-derived exosome in PD. Plasma neural-derived exosomes may serve as a potentially reliable source for accurately reflecting changes in the central nervous system (Goetzl et al., 2015), for they containing jettisoned and potentially toxic forms of α-synuclein or other disease-associated proteins and avoiding interference from blood contamination, systemic inflammation, and potential tumors. In this study, we found the level of ferritin in plasma neural-derived exosomes was significantly higher in patients with PD. It indicated that ferritin in plasma neural-derived exosomes may involve in the occurrence of PD and be a potential biomarker of the disease.

We speculate that the increase in ferritin levels in plasma neural-derived exosomes is due to high iron concentration in neuron in PD. The mechanism for the increased levels of ferritin in the exosome is currently unclear. Ferritin can be secreted through the exosome pathway, and serum ferritin levels typically reflect body iron stores. Ferritin is intricately regulated by cellular iron levels via the iron responsive element-iron regulatory protein system (Gandham et al., 2020). Studies have shown that an increase in high iron concentration in fibroblasts leads to an increase in ferritin secretion through exosomes (Yanatori et al., 2021). Perhaps this can explain the increase of ferritin in plasma neural-derived exosomes found in our study, which needs to be verified by further research.

Iron is delivered by transferrin to all cells in the body through blood vessels. TfR as a membrane protein expressed as a homodimer on the surface of cell, binds to transferrin, mediates cellular iron uptake through internalization, and regulates of oxidative stress. Increased TfR expression facilitates the entry of iron into cells (Kawabata, 2019). The blood of healthy animals contains plenty of TfR+ exosomes (Wallace et al., 2007). Toxin-induced neurodegeneration is associated with progressive increase in the levels and oxidation of transferrin within DA neurons. Such an increase is paralleled by iron deposition. Intracellular levels of transferrin reflect the trafficking mediated by its receptors (TfR) (Wallace et al., 2009; Usman et al., 2018). In this study, the level of TfR in plasma neural-derived exosomes was significantly higher in patients with PD than controls. The ROC analysis performance was found to be only moderate, and logistic analysis indicated it may be an independent risk factor for the disease. The study yielded an intriguing result: a significant positive correlation was observed between ferritin and TfR levels in plasma neural-derived exosomes, suggesting their potential involvement in the mechanism of excessive iron deposition in PD.

In this study, there was no significant correlation association between the levels of ferritin and TfR in plasma neural-derived exosome of PD patients, and the course or severity of the disease. Because it is a cross-sectional study, we cannot tell whether there is a correlation between these biomarkers and disease progression. Our results only suggest that some proteins associated with iron metabolism may play a role in the diagnosis of the disease. Indeed, the roles of different biomarkers in PD are not exactly the same in the aspects of diagnostic potential. For example, neurofilament in blood (Hansson et al., 2017) or CSF (Herbert et al., 2015), only assisted the differentiation of PD, but cannot contribute to the early diagnosis of the disease and the progression of the disease; salivary gland a-syn detection (Tsukita et al., 2019), can only indicate the synucleinopathy, but cannot help the disease differentiation and progression. Among these biomarkers, only CSF a-syn seeding activity detection can reach a > 90% sensitivity and specificity, but the application scenarios have certain limitations and it also cannot evaluate the disease progression (van Rumund et al., 2019; Iranzo et al., 2021). Therefore, the study of biomarkers has been ongoing, not only to be able to serve clinical needs as much as possible, but also to explore the mechanism of disease. There is another limitation that this preliminary study was a small sample size study, and a larger sample size is needed to further support our results.

## Conclusion

Ferritin and TfR levels in plasma neural-derived exosomes show promise as potential biomarkers for PD. TfR maybe the independent risk factor for the disease. However, to validate the significance of these findings and establish any correlation between these biomarkers and disease progression, further studies with larger patient cohorts, long-term following-up, and multi-center study are needed.

## Data availability statement

The original contributions presented in this study are included in the article/supplementary material, further inquiries can be directed to the corresponding authors.

## Ethics statement

The studies involving humans were approved by the Ethics Committees of Fujian Provincial Hospital. The studies were conducted in accordance with the local legislation and institutional requirements. The participants provided their written informed consent to participate in this study.

## Author contributions

Z-TC was responsible for the conception and design of this study, execution of the experimental work, wrote the first draft of the manuscript, and review and critique of the manuscript. C-ZP undertook study design, execution of statistical analysis, execution of experimental work, and the review and critique of the manuscript. L-PL and S-ML organized the research project and reviewed and critiqued the manuscript. Y-ZW organized the research project and review and critiqued the statistical analysis and the manuscript. Z-HZ was responsible for the conception of experiments, execution of experimental work, design and execution of statistical analysis, and the review and critique of the manuscript. All authors contributed to the article and approved the submitted version.

## References

[B1] Ayton S. Diouf I. Bush A. I. Alzheimer’s disease Neuroimaging Initiative (2018). Evidence that iron accelerates Alzheimer’s pathology: A CSF biomarker study. *J. Neurol. Neurosurg. Psychiatry* 89 456–460. 10.1136/jnnp-2017-316551 28939683

[B2] Ayton S. Faux N. G. Bush A. I. (2017). Association of cerebrospinal fluid ferritin level with preclinical cognitive decline in APOE-ε4 carriers. *JAMA Neurol.* 74 122–125. 10.1001/jamaneurol.2016.4406 27893873

[B3] Berardelli A. Wenning G. K. Antonini A. Berg D. Bloem B. R. Bonifati V. (2013). EFNS/MDS-ES/ENS [corrected] recommendations for the diagnosis of Parkinson’s disease. *Eur. J. Neurol.* 20 16–34. 10.1111/ene.12022 23279440

[B4] Calloni S. F. Conte G. Sbaraini S. Cilia R. Contarino V. E. Avignone S. (2018). Multiparametric MR imaging of Parkinsonisms at 3 tesla: Its role in the differentiation of idiopathic Parkinson’s disease versus atypical Parkinsonian disorders. *Eur. J. Radiol.* 109 95–100. 10.1016/j.ejrad.2018.10.032 30527319

[B41] Chawla S. Gulyani S. Allen R. P. Earley C. J. Li X. Van Zijl. P. (2019). Extracellular vesicles reveal abnormalities in neuronal iron metabolism in restless legs syndrome. *Sleep* 42, zsz079. 10.1093/sleep/zsz079 30895312 PMC6612672

[B5] Cohen L. A. Gutierrez L. Weiss A. Leichtmann-Bardoogo Y. Zhang D. L. Crooks D. R. (2010). Serum ferritin is derived primarily from macrophages through a nonclassical secretory pathway. *Blood* 116 1574–1584. 10.1182/blood-2009-11-253815 20472835

[B6] Dehay B. Vila M. Bezard E. Brundin P. Kordower J. H. (2016). Alpha-synuclein propagation: New insights from animal models. *Mov. Disord. Offic. J. Mov. Disord. Soc.* 31 161–168. 10.1002/mds.26370 26347034

[B7] Dickson D. W. Lin W. Liu W. K. Yen S. H. (1999). Multiple system atrophy: A sporadic synucleinopathy. *Brain Pathol.* 9 721–732. 10.1111/j.1750-3639.1999.tb00553.x 10517510 PMC8098455

[B8] Eusebi P. Giannandrea D. Biscetti L. Abraha I. Chiasserini D. Orso M. (2017). Diagnostic utility of cerebrospinal fluid alpha-synuclein in Parkinson’s disease: A systematic review and meta-analysis. *Mov. Disord. Offic. J. Mov. Disord. Soc.* 32 1389–1400. 10.1002/mds.27110 28880418

[B9] Fan K. Jia X. Zhou M. Wang K. Conde J. He J. (2018). Ferritin nanocarrier traverses the blood brain barrier and kills glioma. *ACS Nano* 12 4105–4115. 10.1021/acsnano.7b06969 29608290

[B10] Galvin J. E. Giasson B. Hurtig H. I. Lee V. M. Trojanowski J. Q. (2000). Neurodegeneration with brain iron accumulation, type 1 is characterized by alpha-, beta-, and gamma-synuclein neuropathology. *Am. J. Pathol.* 157 361–368. 10.1016/s0002-9440(10)64548-8 10934140 PMC1850114

[B11] Gandham S. Su X. Wood J. Nocera A. L. Alli S. C. Milane L. (2020). Technologies and standardization in research on extracellular vesicles. *Trends Biotechnol.* 38 1066–1098. 10.1016/j.tibtech.2020.05.012 32564882 PMC7302792

[B12] Goetzl E. J. Boxer A. Schwartz J. B. Abner E. L. Petersen R. C. Miller B. L. (2015). Altered lysosomal proteins in neural-derived plasma exosomes in preclinical Alzheimer disease. *Neurology* 85 40–47. 10.1212/WNL.0000000000001702 26062630 PMC4501943

[B13] Griffiths P. D. Dobson B. R. Jones G. R. Clarke D. T. (1999). Iron in the basal ganglia in Parkinson’s disease. An in vitro study using extended X-ray absorption fine structure and cryo-electron microscopy. *Brain* 122(Pt. 4), 667–673. 10.1093/brain/122.4.667 10219780

[B14] Groger A. Berg D. (2012). Does structural neuroimaging reveal a disturbance of iron metabolism in Parkinson’s disease? Implications from MRI and TCS studies. *J. Neural Transm.* 119 1523–1528. 10.1007/s00702-012-0873-0 22875636

[B15] Gutteridge J. M. (1994). Hydroxyl radicals, iron, oxidative stress, and neurodegeneration. *Ann. N. Y. Acad. Sci.* 738 201–213. 10.1111/j.1749-6632.1994.tb21805.x 7832429

[B16] Hansson O. Janelidze S. Hall S. Magdalinou N. Lees A. J. Andreasson U. (2017). Blood-based NfL: A biomarker for differential diagnosis of parkinsonian disorder. *Neurology* 88 930–937. 10.1212/WNL.0000000000003680 28179466 PMC5333515

[B17] Henrich M. T. Geibl F. F. Lee B. Chiu W. H. Koprich J. B. Brotchie J. M. (2018). A53T-alpha-synuclein overexpression in murine locus coeruleus induces Parkinson’s disease-like pathology in neurons and glia. *Acta Neuropathol. Commun.* 6:39. 10.1186/s40478-018-0541-1 29747690 PMC5946574

[B18] Herbert M. K. Aerts M. B. Beenes M. Norgren N. Esselink R. A. Bloem B. R. (2015). CSF neurofilament light chain but not FLT3 ligand discriminates Parkinsonian disorders. *Front. Neurol.* 6:91. 10.3389/fneur.2015.00091 25999911 PMC4419719

[B19] Iranzo A. Fairfoul G. Ayudhaya A. C. N. Serradell M. Gelpi E. Vilaseca I. (2021). Detection of alpha-synuclein in CSF by RT-QuIC in patients with isolated rapid-eye-movement sleep behaviour disorder: A longitudinal observational study. *Lancet Neurol.* 20 203–212. 10.1016/S1474-4422(20)30449-X 33609478

[B20] Jimenez-Jimenez F. J. Alonso-Navarro H. Garcia-Martin E. Agundez J. A. G. (2021). Biological fluid levels of iron and iron-related proteins in Parkinson’s disease: Review and meta-analysis. *Eur. J. Neurol.* 28 1041–1055. 10.1111/ene.14607 33098743

[B21] Kawabata H. (2019). Transferrin and transferrin receptors update. *Free Radic. Biol. Med.* 133 46–54. 10.1016/j.freeradbiomed.2018.06.037 29969719

[B22] Langkammer C. Schweser F. Krebs N. Deistung A. Goessler W. Scheurer E. (2012). Quantitative susceptibility mapping (QSM) as a means to measure brain iron? A post mortem validation study. *Neuroimage* 62 1593–1599. 10.1016/j.neuroimage.2012.05.049 22634862 PMC3413885

[B23] Martin W. R. Wieler M. Gee M. (2008). Midbrain iron content in early Parkinson disease: A potential biomarker of disease status. *Neurology* 70 1411–1417. 10.1212/01.wnl.0000286384.31050.b5 18172063

[B24] Pan R. Luo S. Huang Q. Li W. Cai T. Lai K. (2022). The Associations of cerebrospinal fluid ferritin with neurodegeneration and neuroinflammation along the Alzheimer’s disease continuum. *J. Alzheimers Dis.* 88 1115–1125. 10.3233/JAD-220002 35754266

[B25] Parnetti L. Gaetani L. Eusebi P. Paciotti S. Hansson O. El-Agnaf O. (2019). CSF and blood biomarkers for Parkinson’s disease. *Lancet Neurol.* 18 573–586. 10.1016/S1474-4422(19)30024-9 30981640

[B26] Postuma R. B. Berg D. Stern M. Poewe W. Olanow C. W. Oertel W. (2015). MDS clinical diagnostic criteria for Parkinson’s disease. *Mov. Disord. Offic. J. Mov. Disord. Soc.* 30 1591–1601. 10.1002/mds.26424 26474316

[B27] Shi M. Kovac A. Korff A. Cook T. J. Ginghina C. Bullock K. M. (2016). CNS tau efflux via exosomes is likely increased in Parkinson’s disease but not in Alzheimer’s disease. *Alzheimers Dement. J. Alzheimers Assoc.* 12 1125–1131. 10.1016/j.jalz.2016.04.003 27234211 PMC5107127

[B28] Shi M. Liu C. Cook T. J. Bullock K. M. Zhao Y. Ginghina C. (2014). Plasma exosomal alpha-synuclein is likely CNS-derived and increased in Parkinson’s disease. *Acta Neuropathol.* 128 639–650. 10.1007/s00401-014-1314-y 24997849 PMC4201967

[B29] Sian-Hulsmann J. Mandel S. Youdim M. B. Riederer P. (2011). The relevance of iron in the pathogenesis of Parkinson’s disease. *J. Neurochem.* 118 939–957. 10.1111/j.1471-4159.2010.07132.x 21138437

[B30] Sugiyama A. Sato N. Kimura Y. Ota M. Maekawa T. Sone D. (2018). MR findings in the substantia nigra on phase difference enhanced imaging in neurodegenerative parkinsonism. *Parkins. Related Disord.* 48 10–16. 10.1016/j.parkreldis.2017.12.021 29279191

[B31] Truman-Rosentsvit M. Berenbaum D. Spektor L. Cohen L. A. Belizowsky-Moshe S. Lifshitz L. (2018). Ferritin is secreted via 2 distinct nonclassical vesicular pathways. *Blood* 131 342–352. 10.1182/blood-2017-02-768580 29074498 PMC5774206

[B32] Tsukita K. Sakamaki-Tsukita H. Tanaka K. Suenaga T. Takahashi R. (2019). Value of in vivo alpha-synuclein deposits in Parkinson’s disease: A systematic review and meta-analysis. *Mov. Disord. Offic. J. Mov. Disord. Soc.* 34 1452–1463. 10.1002/mds.27794 31322768

[B33] Usman W. M. Pham T. C. Kwok Y. Y. Vu L. T. Ma V. Peng B. (2018). Efficient RNA drug delivery using red blood cell extracellular vesicles. *Nat. Commun.* 9:2359. 10.1038/s41467-018-04791-8 29907766 PMC6004015

[B34] van Rumund A. Green A. J. E. Fairfoul G. Esselink R. A. J. Bloem B. R. Verbeek M. M. (2019). alpha-Synuclein real-time quaking-induced conversion in the cerebrospinal fluid of uncertain cases of parkinsonism. *Ann. Neurol.* 85 777–781. 10.1002/ana.25447 30801759 PMC6593725

[B35] Wallace D. F. Summerville L. Subramaniam V. N. (2007). Targeted disruption of the hepatic transferrin receptor 2 gene in mice leads to iron overload. *Gastroenterology* 132 301–310. 10.1053/j.gastro.2006.11.028 17241880

[B36] Wallace D. F. Summerville L. Crampton E. M. Frazer D. M. Anderson G. J. Subramaniam V. N. (2009). Combined deletion of Hfe and transferrin receptor 2 in mice leads to marked dysregulation of hepcidin and iron overload. *Hepatology* 50 1992–2000. 10.1002/hep.23198 19824072

[B37] Wei Z. Li X. Li X. Liu Q. Cheng Y. (2018). Oxidative stress in Parkinson’s disease: A systematic review and meta-analysis. *Front. Mol. Neurosci.* 11:236. 10.3389/fnmol.2018.00236 30026688 PMC6041404

[B38] Yanatori I. Richardson D. R. Dhekne H. S. Toyokuni S. Kishi F. (2021). CD63 is regulated by iron via the IRE-IRP system and is important for ferritin secretion by extracellular vesicles. *Blood* 138 1490–1503. 10.1182/blood.2021010995 34265052 PMC8667049

[B39] Youdim M. B. Riederer P. (1993). The role of iron in senescence of dopaminergic neurons in Parkinson’s disease. *J. Neural Transm. Suppl.* 40 57–67. 8294901

[B40] Zhao Z. H. Chen Z. T. Zhou R. L. Zhang X. Ye Q. Y. Wang Y. Z. (2018). Increased DJ-1 and alpha-synuclein in plasma neural-derived exosomes as potential markers for Parkinson’s disease. *Front. Aging Neurosci.* 10:438. 10.3389/fnagi.2018.00438 30692923 PMC6339871

