# Corrigendum: Evaluation of ferritin and TfR level in plasma neural-derived exosomes as potential markers of Parkinson's disease

**DOI:** 10.3389/fnagi.2023.1355200

**Published:** 2024-01-19

**Authors:** Zhi-ting Chen, Chu-zhui Pan, Xing-lin Ruan, Li-ping Lei, Sheng-mei Lin, Yin-zhou Wang, Zhen-Hua Zhao

**Affiliations:** ^1^Department of Neurology, Fujian Medical University Union Hospital, Fuzhou, Fujian, China; ^2^Shengli Clinical Medical College of Fujian Medical University, Fuzhou, Fujian, China; ^3^Department of Neurology, Fujian Provincial Hospital, Fuzhou, Fujian, China

**Keywords:** biomarker, ferritin, transferrin receptor, exosome, Parkinson's disease

In the published article “Chawla, S., Gulyani, S., Allen, RP., Earley, C.J., Li, X., Van, Zijl. P., et al. (2019). Extracellular vesicles reveal abnormalities in neuronal iron metabolism in restless legs syndrome. *Sleep* 42, zsz079. 10.1093/sleep/zsz079” was not cited in the article. The citation has now been inserted in introduction, Paragraph 5 and should read:

“Studies of iron and iron-related proteins levels in cerebrospinal fluid (CSF), serum, plasma, and urine have shown conflicting results. Meta-analyses of these studies have revealed that CSF and serum/plasma ferritin and transferrin concentrations did not differ significantly between PD patients and controls (Jimenez-Jimenez et al., 2021). However, recent studies have shown that exosomes were a pathway for neurons to divert proteins from neurons into the CSF or into the peripheral blood via the blood-brain barrier (Shi et al., 2014). Exosomal a-synuclein in neural-derived blood exosomes was found to be increased in patients with PD (Shi et al., 2016). Chalwa's group studied the iron homeostasis in restless legs syndrome, and found that the patients had higher levels of total ferritin and heavy-chain ferritin, and similar levels of TfR in serum neural-derived exosomes (Chawla et al., 2019). To investigate the mechanism of iron deposition and to identify potential novel biomarkers for PD, this study evaluated the levels of ferritin and TfR level in plasma neural-derived exosomes using enzyme-linked immunosorbent assay (ELISA).”

In the published article, there was an error. Chawla's team has made pioneering work on the study of iron metabolism in blood neuro-exosomes, although the patients in their study were RLS, but not PD. There are some of the expressions in our original article are not rigorous enough.

A correction has been made to abstract, introduction. This sentence previously stated:

“While the blood of healthy animals contains a plentiful supply of TfR positive exosomes, no studies have examined ferritin and TfR in plasma neuralderived exosomes.”

The corrected sentence appears below:

“While the blood of healthy animals contains a plentiful supply of TfR positive exosomes, rare study has examined ferritin and TfR in plasma neural-derived exosomes in PD.”

The other correction has been made to introduction, Paragraph 5. This sentence previously stated:

“To investigate the mechanism of iron deposition and to identify potential novel biomarkers for PD, this study evaluated the levels of ferritin and TfR level in plasma neural-derived exosomes using enzyme-linked immunosorbent assay (ELISA).”

The corrected sentence appears below:

“Chalwa's group studied the iron homeostasis in restless legs syndrome, and found that the patients had higher levels of total ferritin and heavy-chain ferritin, and similar levels of TfR in serum neural-derived exosomes (Chawla et al., 2019). To investigate the mechanism of iron deposition and to identify potential novel biomarkers for PD, this study evaluated the levels of ferritin and TfR level in plasma neural-derived exosomes using enzyme-linked immunosorbent assay (ELISA).”

In the published article, there was an error in “Figure 3. Comparation of ferritin and TfR in PD and controls, and ROC analysis of biomarkers for PD diagnosis” as published. In Figure 3A and 3B, “PD” and “control” was reversed by mistake. The corrected “Figure 3. Comparation of ferritin and TfR in PD and controls, and ROC analysis of biomarkers for PD diagnosis” and its caption appear below. In Figure 3A and 3B, “PD” and “control” swapped positions.

**Figure 3:**
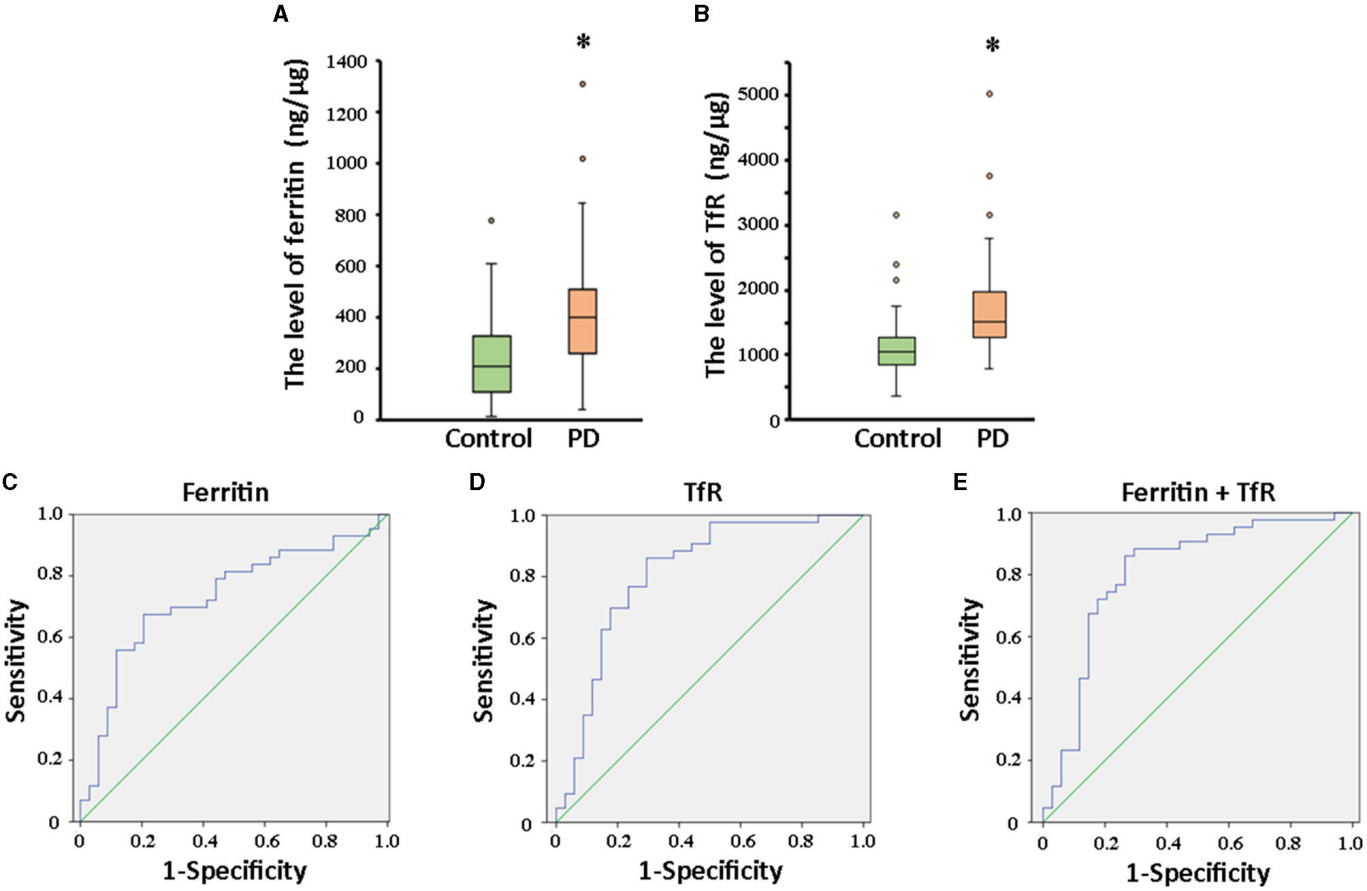
Comparation of ferritin and TfR in PD and controls, and ROC analysis of biomarkers for PD diagnosis. The box plots showed the comparison of the levels of ferritin **(A)** and TfR **(B)** in the two groups. ^*^, *P* < 0.01. In the whole cohort, ferritin in plasma neural-derived exosomes provided an AUC of 0.73 (sensitivity = 67.4%, specificity = 79.4%) for PD versus controls **(C)**. TfR performed similarly (AUC = 0.812, sensitivity = 86.0%, specificity = 70.6%) in the whole cohort **(D)**. And, the combination of ferritin and TfR did not obviously improve the performance with AUC of 0.808 (sensitivity = 86.0%, specificity = 73.5%) **(E)**.

The authors apologize for this error and state that this does not change the scientific conclusions of the article in any way. The original article has been updated.
